# Supplementary material for: The prevalence of mental health and addiction concerns and factors associated with depression and anxiety during the COVID-19 pandemic in Ontario, Canada: A cross-sectional study
Source: PLoS One. 2024 Jun 12;19(6):e0305229. doi: 10.1371/journal.pone.0305229 (PMC11168667; doi:10.1371/journal.pone.0305229)
Supplement: Attachment — Submitted filename: Response to Reviewers.docx [file pone.0305229.s001.docx]

December 19, 2023

**Re: PONE-D-23-30089 - The prevalence of mental health and addiction concerns and factors associated with depression and anxiety during the COVID-19 pandemic in Ontario, Canada: A cross-sectional study**

Dear Dr. Chenette and Editorial Board,

Thank you very much for providing us with the opportunity to revise and resubmit our manuscript entitled, “The prevalence of mental health and addiction concerns and factors associated with depression and anxiety during the COVID-19 pandemic in Ontario, Canada: A cross-sectional study” - Manuscript ID: PONE-D-23-30089.

We appreciate your providing us with the reviewers’ helpful comments and suggestions. Please find our itemized, point-by-point response to each item beside the reviewers’ original comments along with page numbers and manuscript sections for each comment in the new clean version of the revised manuscript, as requested. We also attached a version of the manuscript text file in MS Word with revisions identified with track changes.

We verify that all information and materials in the manuscript are original. This work was supported by the Canadian Institutes of Health Research (AL, SK, RM; grant number UIP-178831) and the Hurvitz Brain Sciences Summer Student Research Program (OC) through the Sunnybrook Research Institute. The funders had no role in study design, data collection and analysis, decision to publish, or preparation of the manuscript. There was no additional external funding received for this study.

Data collected for this study, including individual participant data and a data dictionary defining each field in the set, are not publicly available because they contain potentially identifying and sensitive patient information, and participants did not consent to the sharing of data on public repositories. Upon request from investigators, data can be made available. Such requests should include a study protocol with clear hypotheses, and be sent to the corresponding author, which will then be reviewed by the study principal investigator and investigator team. If the hypothesis is valid and complies with the authorizations for this study, a data transfer agreement will be developed prior to transfer of de-identified data.

We would like to thank you and the reviewers for their careful review of our manuscript. We believe that it is much improved because of this input.

Yours sincerely,

Oswin Chang, BHSc

and

Roula Markoulakis, PhD

Sunnybrook Research Institute

**PONE-D-23-30089 - The prevalence of mental health and addiction concerns and factors associated with depression and anxiety during the COVID-19 pandemic in Ontario, Canada: A cross-sectional study**

Authors’ Response to Reviewers:

We have numbered and itemized each reviewer comment in the left column below and provided a response to each comment in the right column. We also refer to the changes made according to the reviewers’ requests, with page numbers and sections as they appear in the revised manuscript.

We also attest that we have submitted: (1) our revised manuscript text file in Word, with revisions identified with track changes, as well as (2) a clean version without track changes.

| Reviewer Comment | Author Responses |
| --- | --- |
| **Reviewer 1** | We thank Reviewer 1 for their helpful and insightful comments on our manuscript. We have revised accordingly for each item. Please see details below. |
| 1. It is necessary adequately define the objective of the study, what specific result you want to present and redo the analysis in that sense. What does this article contribute to? | The aim of the study has been clarified for readers by using clearer wording. Moreover, the Introduction has been expanded to highlight what this study contributes to (please see comment 2 below).  **Introduction, pages 4-5, paragraph 4**  “This study’s objective was to identify the prevalence of depression, anxiety, and substance use problems in the general population of Ontario, Canada at a time point nearly two years into the pandemic. Furthermore, we sought to determine which demographic characteristics and social determinants of health were independently associated with symptoms of depression or anxiety.” |
| 2. Introduction - It covers many topics without delving into any of them, prevalence, predictors, disparities…. It is not clear what he calls “mental health concerns”. | Additional prose has been added into the Introduction to provide further elaboration on the topics discussed, including prevalence and predictors. We have removed mention of disparities as that is out of scope for the current study. Furthermore, mental health concerns specifically refer to depression and anxiety, and we have replaced “mental health concerns” where applicable.  **Introduction, pages 3-4, paragraphs 1-2**  “Globally, depression and anxiety have been estimated to affect 4.4% and 3.6% of the population, respectively [1]. In the early months of the COVID-19 pandemic, increased rates of screening positive for depression and anxiety were well-documented in general population samples, with some prevalence estimates placing depression at 28% and anxiety at 33% [2]. These findings may be unsurprising considering the implementation of various public health measures during the pandemic, from lockdowns to social distancing, which may have upended many lives due to school closures [3], increased caregiving and household responsibilities for parents [4], healthcare services being cut [5], and greater financial difficulties [6]. However, some subsequent studies suggested that screening positive for depression and anxiety increased as the pandemic progressed [7,8], while others suggested consistent rates or decreases [9,10] These heterogeneous findings may be due to varying pandemic severities in different countries, the type/length of societal restrictions, the timing of measurements, methodological differences, availability of vaccines, or the populations themselves [11–13]. Thus, a more up-to-date prevalence of these outcomes from various global jurisdictions is needed to address specific regional needs.  Various factors have been found to be associated with symptoms of depression or anxiety pre-pandemic, such as female gender, younger age, marital challenges, chronic health conditions, poor familial relationships, financial difficulties, unemployment, and family history [14–18]. During the pandemic, some studies reported experiencing symptoms of depression or anxiety was found to be related to younger age, female gender, ethnic minority status, low socioeconomic status (SES), living situation, and non-married marital status [19–22]. However, not all of these factors were found to be significant in all studies, which suggests that additional contextual influences may be an important consideration, so further exploration into people’s experiences with the pandemic itself is warranted. Considering factors such as access to healthcare services, social support, or fear are related to mental health and have been directly impacted due to the pandemic or associated public health measures [5,23,24], there is a need to determine how they relate to and influence depression or anxiety in the pandemic context. Thus, understanding which of these factors and what other sociodemographic characteristics or experiences linked to the pandemic are associated with the presence of depressive and anxiety symptoms at a later timepoint in the pandemic is necessary because as pandemic conditions change, decision-makers require more data to better distribute mental health and/or addiction (MHA) resources.” |
| 3. Methods - It is not described, everything refers to another document, it is not possible for the reader to understand the basic source of information or methodology with what is provided in the section.  1) It seems to me that there was no reflection on the results to be presented, there is no robust conceptual or theoretical presentation.  It is not defined what they call MHA or pandemic related outcomes. | a) Our rationale for selecting the variables that we did was based on previous research that had identified these factors as related to depression or anxiety (1-3). However, not all studies found these factors to be statistically significant, which suggests that varying populations or people’s experience with the pandemic may influence results. Thus, we believed it was important to re-examine factors that had been previously examined in addition to other variables not previously explored that were directly affected by the pandemic and associated public health measures (e.g., access to healthcare services, social supports, fear of COVID-19 (4-6)) to ensure that findings would be applicable specifically for the population of Ontario, Canada while still generalizable to other settings. (1) Betini GS, Hirdes JP, Adekpedjou R, Perlman CM, Huculak N, Hébert P. Longitudinal Trends and Risk Factors for Depressed Mood Among Canadian Adults During the First Wave of COVID-19. Front Psychiatry. 2021;12:666261. Published 2021 Jul 16. doi:10.3389/fpsyt.2021.666261 (2) Czeisler MÉ, Lane RI, Petrosky E, et al. Mental Health, Substance Use, and Suicidal Ideation During the COVID-19 Pandemic - United States, June 24-30, 2020. MMWR Morb Mortal Wkly Rep. 2020;69(32):1049-1057. Published 2020 Aug 14. doi:10.15585/mmwr.mm6932a1 (3) Ettman CK, Abdalla SM, Cohen GH, Sampson L, Vivier PM, Galea S. Prevalence of Depression Symptoms in US Adults Before and During the COVID-19 Pandemic. JAMA Netw Open. 2020;3(9):e2019686. Published 2020 Sep 1. doi:10.1001/jamanetworkopen.2020.19686 (4) World Health Organization. COVID-19 disrupting mental health services in most countries, WHO survey. World Health Organization 2020. https://www.who.int/news/item/05-10-2020-covid-19-disrupting-mental-health-services-in-most-countries-who-survey (accessed October 14, 2021). (5) Luo F, Ghanei Gheshlagh R, Dalvand S, Saedmoucheshi S, Li Q. Systematic Review and Meta-Analysis of Fear of COVID-19. Front Psychol. 2021;12:661078. Published 2021 Jun 11. doi:10.3389/fpsyg.2021.661078 (6) Banerjee D, Rai M. Social isolation in Covid-19: The impact of loneliness. Int J Soc Psychiatry. 2020;66(6):525-527. doi:10.1177/0020764020922269 b) MHA has been defined under the header “MHA Outcomes” to specifically include depression, anxiety, and substance use (tobacco, alcohol, cannabis, opioids, other) as well as how they were measured.   Our pandemic-related outcomes include COVID-19 fear, impact of pandemic on mental health, social support, socioeconomic status (SES), and MHA service needs. We have included a description in the Method section under “Pandemic-related Outcomes” defining the variables.  **Method, pages 6-7, paragraphs 3-9**  “**MHA Outcomes**  Depression and Anxiety: The Diagnostic and Statistical Manual of Mental Disorders, Fifth Edition, (DSM-5) Self-Rated Level 1 Cross-Cutting Symptom Measure—Adult is a self-reported measure that assesses mental health symptoms from 13 psychiatric domains over the past two weeks [31]. However, only the Depression and Anxiety domains were used for this study. There are two and three items, respectively, from the Depression and Anxiety domains, and all items are scored on a 5-point (0-4) scale with higher scores suggesting greater problems in that domain [31]. If any item receives a score ≥2, the participant screens positive in that psychiatric domain and should be further evaluated.  Substance Use: Participants also completed the Alcohol, Smoking and Substance Involvement Screening Test (ASSIST) Version 3.0, which is an 8-item measure assessing risky or problematic use for 10 substance categories [32,33]. Aside from tobacco products, alcoholic beverages, cannabis, and opioids, remaining substances were classified under the “other substances” category (e.g., cocaine, amphetamine type stimulants, inhalants, sedatives/sleeping pills, hallucinogens, and other) for this study. Specific substance involvement (SSI) scores 0-3 indicate low risk, 4-26 signifies moderate risk, and ≥27 is high risk. The exception is alcohol, whose scoring cutoffs (from low to high risk) are 0-10, 11-26, and ≥27 [32,33]. Scores were classified into low and moderate/high risk for this study.  **Pandemic-related Outcomes**  COVID-19 Fear: Participants commented on their fear of catching COVID-19 themselves or someone close to them contracting it in the past week. Options included not worried, slightly, moderately, very, and extremely worried. Responses were dichotomized as not worried versus worried.  Impact of Pandemic on Mental Health: Participants also rated how the pandemic has affected their mental health. Options included unchanged, a little better/worse, a lot better/worse, very much better/worse. Responses were categorized into worse versus no change/better.  Social Support: Satisfaction with the availability of natural social supports (e.g., friends, family, romantic partners, community groups, coworkers, pets, etc.) since the onset of the pandemic was rated by participants as neither (dis)satisfied, (dis)satisfied, very (dis)satisfied, or extremely (dis)satisfied. Support was dichotomized into satisfied and neither/dissatisfied.  SES: The MacArthur Scale of Subjective Social Status-Adult Version was used to assess SES [34]. Participants completed two 10-point (1-10) slider scales, one requiring retrospective recall for the months just preceding the pandemic and one for during the pandemic, asking them to compare their own income, education, and jobs to those around them. A score of 1 was worst off and 10 was best off. The change in SES was calculated as the difference between the pandemic and pre-pandemic period, and results were classified as worse off and no change/better off.  MHA Service Needs: Thoughts of using MHA services since the pandemic was declared were reported by participants. Services encompassed but were not limited to sessions with healthcare providers, crisis supports, residential treatment, support groups, and MHA programs. Responses were grouped into having needs unmet (thought about accessing services but did not) and needs met (not needing services/needing and accessing services).” |
| 4. Methods - 2) how they model the socioeconomic condition, to adequately isolate the symptoms from what is attributable to the experience with the COVID pandemic. | Please see comment 3b above for how SES was defined. We asked participants to comment on their SES in relation to the pandemic in order to attribute any changes in SES to the pandemic. While we recognize this method cannot provide certainty due to other potentially competing factors, our goal was to look at associations between SES and depression/anxiety rather than determine causality. A line has been added to the limitations to comment on our focus on associations and inability to assign causality.  **Discussion, page 26, paragraph 5**  “Lastly, the cross-sectional design of the study did not allow for a longitudinal investigation, meaning this study can provide insight into associations only and cannot comment on or assign causality.” |
| 5. Methods - Omissions with potentially serious biases in the prevalence measurement:  • They do not include if people already had a diagnosis and/or received treatment for the mental health condition, then, prevalence of what the survey measures? people with symptoms and not treated, or treated inappropriately?  • The presence of symptoms of depression and anxiety represents 35.7% of the study population, and authors omits to analyze this population!! | We thank the reviewer for raising these points.   a) The inclusion criteria for the study were: >18 years of age, living in the province of Ontario, Canada, and being registered with Delvinia’s AskingCanadians respondent panel. The exclusion criteria was: respondent’s quota full (based on a priori quotas for age, gender, and region). The study sampled from the general population and not based on any specific diagnosis or treatment plan. Thus, the prevalences reported reflect that of the general population. The inclusion/exclusion criteria listed above have been added to the Method section.   However, we did ask about MHA service needs (described in comment 3b), which was included in the logistic regression analyses and is currently being investigated in other manuscripts examining service use during the pandemic.  **Method, page 5, paragraph 1**  “The inclusion criteria for the study were: >18 years of age, living in the province of Ontario, Canada, and being registered with Delvinia’s AskingCanadians respondent panel. The exclusion criteria was: respondent’s quota full (based on a priori quotas for age, gender, and region).”   b) We have removed mention of the statistic on depression and anxiety comorbidity so as to not distract readers from our objective which was to analyze depression and anxiety independently. While depression and anxiety are frequently comorbid and share some similarities, we modelled them separately because depression and anxiety are distinct cognitive states with different manifestations and effects on functioning, which warrant independent analyses in the context of this study (1-3). (1) Eysenck MW, Fajkowska M. Anxiety and depression: toward overlapping and distinctive features. *Cogn Emot*. 2018;32(7):1391-1400. doi:10.1080/02699931.2017.1330255 (2) Kreitler S. The meaning profiles of anxiety and depression: similarities and differences in two age groups. *Cogn Emot*. 2018;32(7):1499-1513. doi:10.1080/02699931.2017.1311248 (3) Drost J, Van der Does AJ, Antypa N, Zitman FG, Van Dyck R, Spinhoven P. General, Specific and Unique Cognitive Factors Involved in Anxiety and Depressive Disorders. *Cognit Ther Res*. 2012;36(6):621-633. doi:10.1007/s10608-011-9401-z |
| 6. Methods - Using the word predictors is wrong as it comes from a cross-sectional survey | We thank the reviewer for their attention to detail. The term “predictor” and its variations have been replaced with “association” and its variations. |
| 7. Methods - Why ruin all the work of collecting a large sample by dividing the statistical analysis of the sample into two parts. Why a separate regression model for anxiety and another for depression, what justification does this strategy have, and again why does it not model the depression-anxiety comorbidity? | Please see comment 5b for our explanation on the removal of the depression-anxiety comorbidity datum and why separate models were created. |
| 8. Methods - The regression models seem poorly worked to me, examples:  • What was the point of seeing in Table 1 that there are differentiated patterns by age, if the multivariate model includes the age variable as a linear continuous, without any type of adjustment.  • What the variables Fear of covid, unmet MHA service needs mean. | We thank the reviewer for allowing us to clarify.   a) The age groups (18-34, 35-54, and ≥55) found in Table 1 represent the age quotas used to sample the population. In the multivariate models, age was analyzed as a continuous variable because there are many issues with using categorized forms of continuous data, such as reduced statistical power and creating boundaries that do not exist, leading to false impressions of threshold effects (i.e., these age groups are arbitrary and might give the false impression that a 34 year old has more in common with an 18 year old than a 35 year old) (1, 2). (1) Altman DG, Royston P. The cost of dichotomising continuous variables. *BMJ*. 2006;332(7549):1080. doi:10.1136/bmj.332.7549.1080 (2) Royston P, Altman DG, Sauerbrei W. Dichotomizing continuous predictors in multiple regression: a bad idea. *Stat Med*. 2006;25(1):127-141. doi:10.1002/sim.2331 b) Please see point 3b above for definitions of COVID-19 fear and MHA service needs. |
| 9. Results - Table 1: It is necessary to reflect on what is presented  - How useful are the columns (not depression and not anxiety)?  - Why population with anxiety and depression symptoms are no presented?  - I think these columns should be contrast: Total population, Pop with symptoms of depression, Pop with symptoms of anxiety, Pop with symptoms of both, and Pop without symptoms | a) We have removed the columns for participants with no depression as well as no anxiety. b) Please refer to comment 5b for rationale on why we are no longer reporting that subgroup. c) The new table columns now only include total population, population with depression, and population with anxiety as we are not examining participants with both depression and anxiety. |
| 10. Results - Table 2: I do not understand this table,  - Why not present levels or severity of symptoms instead of presenting averages.  - How useful is it to compare the average response of the non-depressed population with depression, if the measuring instrument to classify in column as in row was the same | a) The Diagnostic and Statistical Manual of Mental Disorders, Fifth Edition, (DSM-5) Self-Rated Level 1 Cross-Cutting Symptom Measure—Adult was used to assess symptoms of depression and anxiety. There are two and three items, respectively, from the depression and anxiety domains, and all items are scored on a 5-point (0-4) scale with higher scores suggesting greater problems in that domain (1). If any item receives a score ≥2, the participant screens positive in that psychiatric domain and should be further evaluated. Since this measure dichotomizes outcomes as positive or negative in a domain, there are no severity levels to present. Thus, we opted instead to present the average score of participants. (1) Narrow WE, Clarke DE, Kuramoto SJ, et al. DSM-5 field trials in the United States and Canada, Part III: development and reliability testing of a cross-cutting symptom assessment for DSM-5. *Am J Psychiatry*. 2013;170(1):71-82. doi:10.1176/appi.ajp.2012.12071000 b) We have made the table clearer by adding the term “domain” to the DSM-5 score rows and removing depression scores for patients screening positive for anxiety and vice versa. |
| 11. Results - Table 3 and 4: It seems absurd to me to divide the analysis into two models, the study variable is mental health at the population level. It is necessary to reflect on how to properly model | Please refer to comment 1 for clarification of study objective, which was examining depression and anxiety at the population level, not mental health in general.  Additionally, please refer to comment 5b for rationale on why separate models were constructed for depression and anxiety. |

| Reviewer Comment | Author Responses |
| --- | --- |
| **Reviewer 2** | We thank Reviewer 2 for their helpful and insightful comments on our manuscript. We have revised accordingly for each item. Please see details below. |
| 12. The study design is cross-sectional and the attribution of causality with the pandemic has important limitations inherent to the data. Therefore, it is important that the authors point out more clearly that the increase in the occurrence of mental health and addiction concerns may be due to the pandemic or due to greater visibility of mental health issues (better and greater diagnostic coverage). | We thank the reviewer for raising this point. A sentence has been added to the Discussion to indicate more clearly that the increase in the occurrence of mental health and addiction concerns may be due to the pandemic or due to greater visibility of mental health issues.  **Discussion, page 23, paragraph 2**  “Concurrently, increased prevalence rates in the present study may be due to increased awareness and visibility surrounding mental health issues during the pandemic.” |
| 13. From a methodological point of view, the statistical analysis did not take advantage of the possibility of exploring contextual effects that seem to have had an impact on the findings. See table 1 for the differences between Southwestern Ontario, Eastern Ontario and Central Ontario. It would be interesting to know the results of a multilevel analysis, since the findings on the protective association of social support are suggestive of differential social processes between the regions. | While it may be possible that social processes may have differed between regions, the COVID-19 public health response was coordinated by the Government of Ontario to ensure consistent implementation of health measures throughout the province. As a result, we do not believe different rates of screening positive for depression or anxiety are a function of the region themselves. Rather, previous studies have shown how factors such as age, gender, ethnicity, etc. are related to depression or anxiety (1-3). We believe the differences in depression and anxiety found in certain regions may be due to these participant characteristics or people’s experiences during the pandemic (e.g., social support, service needs, etc.), which is why these variables were included in models and not geographic region.   (1) Betini GS, Hirdes JP, Adekpedjou R, Perlman CM, Huculak N, Hébert P. Longitudinal Trends and Risk Factors for Depressed Mood Among Canadian Adults During the First Wave of COVID-19. Front Psychiatry 2021;12:666261. https://doi.org/10.3389/fpsyt.2021.666261. (2) Czeisler MÉ, Lane RI, Petrosky E, Wiley JF, Christensen A, Njai R, et al. Mental Health, Substance Use, and Suicidal Ideation During the COVID-19 Pandemic - United States, June 24-30, 2020. MMWR Morb Mortal Wkly Rep 2020;69:1049–57. https://doi.org/10.15585/mmwr.mm6932a1. (3) Ettman CK, Abdalla SM, Cohen GH, Sampson L, Vivier PM, Galea S. Prevalence of Depression Symptoms in US Adults Before and During the COVID-19 Pandemic. JAMA Netw Open 2020;3:e2019686. https://doi.org/10.1001/jamanetworkopen.2020.19686 |

We wanted to thank the editor and all of the reviewers again for their thoughtful review of our manuscript and for helping to strengthen it further.
