# Supplementary material for: The prevalence of mental health and addiction concerns and factors associated with depression and anxiety during the COVID-19 pandemic in Ontario, Canada: A cross-sectional study
Source: PLoS One. 2024 Jun 12;19(6):e0305229. doi: 10.1371/journal.pone.0305229 (PMC11168667; doi:10.1371/journal.pone.0305229)
Supplement: Attachment — Submitted filename: Response to Reviewers.docx [file pone.0305229.s002.docx]

May 10, 2024

**Re: PONE-D-23-30089R1 - The prevalence of mental health and addiction concerns and factors associated with depression and anxiety during the COVID-19 pandemic in Ontario, Canada: A cross-sectional study**

Dear Dr. Chenette and Editorial Board,

Thank you very much for providing us with the opportunity to revise and resubmit our manuscript entitled, “The prevalence of mental health and addiction concerns and factors associated with depression and anxiety during the COVID-19 pandemic in Ontario, Canada: A cross-sectional study” - Manuscript ID: PONE-D-23-30089R1.

We appreciate your providing us with the reviewers’ helpful comments and suggestions. In light of these comments, we have redone the analyses so that those with depression only, anxiety only, both, and neither are examined separately. The manuscript has also been updated to account for these new analyses. Please find our itemized, point-by-point response to each of the reviewers’ comments along with page numbers and manuscript sections for each comment in the new clean version of the revised manuscript, as requested. We also attached a version of the manuscript text file in MS Word with revisions identified with track changes.

We would like to thank you and the reviewers for their careful review of our manuscript. We believe that it is much improved because of this input.

Yours sincerely,

Oswin Chang, BHSc

and

Roula Markoulakis, PhD

Sunnybrook Research Institute

**PONE-D-23-30089R1 - The prevalence of mental health and addiction concerns and factors associated with depression and anxiety during the COVID-19 pandemic in Ontario, Canada: A cross-sectional study**

Authors’ Response to Reviewers:

We have numbered and itemized each reviewer comment in the left column below and provided a response to each comment in the right column. We also refer to the changes made according to the reviewers’ requests, with page numbers and sections as they appear in the revised manuscript.

We also attest that we have submitted: (1) our revised manuscript text file in Word, with revisions identified with track changes, as well as (2) a clean version without track changes.

| Reviewer Comment | Author Responses |
| --- | --- |
| **Reviewer 1** | We thank Reviewer 1 for their helpful and insightful comments on our manuscript. We have revised accordingly for each item. Please see details below. |
| 1. Although the authors responded to each observation, substantial aspects were left unaddressed. The study could provide information on the prevalence of depression and anxiety symptoms, 2 years after the appearance of COVID-19 and contact restrictions. However, with the information added by the authors, in this second review I doubt whether the study is representative of the target population, since they mentioned that it is a non-random sample.  The methodological description of population selection remains incomplete. Crucial aspects to understand how the sample was selected were omitted. In the response they showed that the sampling was a non-random sampling by quotas, this aspect totally changes the external validity of the study, and it would be necessary for the authors to show simple evidence of the representativeness of the sample, in the same document, and not just reference other documents.  Another aspect about the selection is that they report that they were participants registered in “Delvinia’s AskingCanadians responder”, without explaining in the slightest or making any reference to it. | We appreciate the reviewer giving us the opportunity to expand on our methodology. Delvinia is a reputable research firm, whose respondent panel, AskingCanadians, was commonly used among research collaborations and projects that took place during the pandemic that were critical for developing a national profile and understanding the impacts the pandemic was having on Canadians (1-4). This respondent panel has also been used by the Government of Canada for other research purposes (5). Details about Delvinia have been added to the Methods.  **Methods, page 6, paragraphs 1-2**  “The inclusion criteria for the study were: ≥18 years of age, living in the province of Ontario, Canada, and being registered with Delvinia’s AskingCanadians respondent panel. The exclusion criteria was: respondent’s quota full (based on *a priori* quotas for age, gender, and region). The interlocking quota’s purpose was to establish representativeness based on age and gender for Ontario residents with differences under five percent of the mean or five percentage points total, as applicable. Eligible participants were randomly contacted.  The AskingCanadians panel consists of more than 1 million Canadians. The panel is regularly benchmarked against data from Statistics Canada to ensure representativeness, and more than 500 sociodemographic measures are collected to help ensure panelists meet researchers’ needs (6).”  We also thank the reviewer for raising the point about showing representativeness. This information has now been added to the Results section.  **Results, page 10, paragraph 2**  “According to census data from Statistics Canada for 2022, the sample was representative based on age and sex. The mean age of participants was 47.9 years (within 5% of the mean age of those ≥18 years old living in Ontario, which is 48.3 years), and our sample was comprised of 49.3% male and 49.3% female participants (both within 5% of the sex proportions of Ontario, which is 49.1% male and 50.9% female) (7). Despite using regional quotas, less populated regions (outside of Toronto – Ontario’s largest city) were purposely oversampled so that in each area, the known prevalence of depression could be estimated within a 5% margin of error based on sufficient sample size so that appropriate conclusions could be reached (8).”  We also recognize that this oversampling may reduce the representativeness of our findings. A line has been added to the limitations to discuss this potential issue.  **Discussion, pages 30-31, paragraph 5**  “Furthermore, populations anticipated to have lower response rates (such as in Northern Ontario which is more rural) were oversampled so that the prevalence of mental health symptoms could be estimated within a 5% margin of error. However, this oversampling may also reduce the representativeness of our results, and there may also have been self-selection bias, so caution should be exercised when interpreting these findings.”   (1) Courdi C, Ramazan Ali S, Pelletier-Dumas M, et al. How level of understanding and type of used sources relate to adherence to COVID-19 public health measures in Canada. *Sci Rep*. 2023;13(1):13065. Published 2023 Aug 11. doi:10.1038/s41598-023-38824-0 (2) Mougharbel F, Sampasa-Kanyinga H, Heidinger B, Corace K, Hamilton HA, Goldfield GS. Psychological and Demographic Determinants of Substance Use and Mental Health During the COVID-19 Pandemic. *Front Public Health*. 2021;9:680028. Published 2021 Jun 25. doi:10.3389/fpubh.2021.680028 (3) Miconi D, Levinsson A, Frounfelker RL, Li ZY, Oulhote Y, Rousseau C. Cumulative and independent effects of experiences of social adversity on support for violent radicalization during the COVID-19 pandemic: the mediating role of depression. *Soc Psychiatry Psychiatr Epidemiol*. 2022;57(6):1221-1233. doi:10.1007/s00127-022-02244-8 (4) Elton-Marshall T, Wells S, Jankowicz D, et al. Multiple COVID-19 Risk Factors Increase the Likelihood of Experiencing Anxiety Symptoms in Canada. *Can J Psychiatry*. 2021;66(1):56-58. doi:10.1177/0706743720949356 (5) Government of Canada Publications. *LGBTQ2 Experiences with Stigma and Discrimination in the Workplace*. Updated March 21, 2020. Accessed February 12, 2024. https://publications.gc.ca/collections/collection_2020/bcp-pco/CP22-188-2020-eng.pdf (6) AskingCanadians. Delvinia. Accessed February 16, 2024. https://www.delvinia.com/solutions/askingcanadians/. (7) Statistics Canada. Table 17-10-0005-01 Population estimates on July 1st, by age and sex. doi: 10.25318/1710000501-eng (8) Levitt AJ, Boyle MH, Joffe RT, Baumal Z. Estimated prevalence of the seasonal subtype of major depression in a Canadian community sample. *Can J Psychiatry*. 2000;45(7):650-654. doi:10.1177/070674370004500708 |
| 2. Another important aspect is that, given the observation that the analysis "independently" analyzes the population with two of the health conditions, the authors only respond "because it is of interest to them." However, they do not support this assumption of independence on background, nor do they do any statistical treatment to show what they are talking about. I find it questionable that they show blindness to a large proportion of the population who report both health conditions. | We have now included a multinomial logistic regression that includes the four following groups: depression only, anxiety only, both depression and anxiety, and neither. Furthermore, additional information has been added to the Results section along with Table 3 for these specific findings. The manuscript has also been updated to reflect the addition of this information.  **Results, pages 20-21, paragraphs 2-4**  “Compared to the neither depression nor anxiety group, the depression only group was more likely to be BIPOC (Black, Indigenous, and People of Color) (OR = 1.35, 95% CI = 1.05-1.74, p = .02), have moderate/high risk levels of alcohol use (OR = 1.41, 95% CI = 1.03-1.93, p = .03), have negative change in mental health since the pandemic started (OR = 4.07, 95% CI = 3.22-5.14, p < .001), and have unmet MHA service needs (OR = 2.19, 95% CI = 1.61-2.97, p < .001). Satisfaction with social support decreased the likelihood of being in the depression only group (OR = 0.52, 95% CI = 0.44-0.61, p = .002) as compared to the neither depression nor anxiety group. No other independent variables were significant.  The anxiety only group was more likely to be in the 18-34 (OR = 2.87, 95% CI = 2.09-3.94, p < .001) and 35-48 age groups (OR = 1.88, 95% CI = 1.39-2.56, p < .001), be female (OR = 1.64, 95% CI = 1.34-2.02, p < .001), have moderate/high risk levels of alcohol use (OR = 1.53, 95% CI = 1.15-2.05, p = .004), have COVID-19 infection fear (OR = 1.45, 95% CI = 1.16-1.81, p < .001), have negative change in mental health since the pandemic started (OR = 3.12, 95% CI = 2.52-3.85, p < .001), and have unmet MHA service needs (OR = 3.01, 95% CI = 2.32-3.91, p < .001) compared to the neither depression nor anxiety group. No other independent variables were significant.  Compared to the group with neither depression nor anxiety, the group screening positive for both depression and anxiety was more likely to be in the 18-34 (OR = 3.06, 95% CI = 2.38-3.94, p < .001), 35-48 (OR = 2.10, 95% CI = 1.65-2.67, p < .001), and 49-61 (OR = 1.69, 95% CI = 1.34-2.13, p < .001) age groups; be female (OR = 1.27, 95% CI = 1.09-1.49, p = .003); have moderate/high risk levels of tobacco (OR = 1.40, 95% CI = 1.13-1.72, p = .002), alcohol (OR = 1.98, 95% CI = 1.59-2.47, p < .001), and cannabis (OR = 1.80, 95% CI = 1.43-2.27, p < .001) use; have COVID-19 infection fear (OR = 1.53, 95% CI = 1.29-1.82, p < .001); have negative change in mental health since the pandemic started (OR = 6.91, 95% CI = 5.83-8.20, p < .001); have negative change in SES since the pandemic started (OR = 1.44, 95% CI = 1.21-1.71, p < .001); and have unmet MHA service needs (OR = 5.32, 95% CI = 4.33-6.55, p < .001). Positive screening for symptoms of both depression and anxiety was less likely to occur for those more satisfied with social supports during the pandemic (OR = 0.71, 95% CI = 0.57-0.88, p < .001) as compared to the neither depression nor anxiety group. No other independent variables were significant.”  **Discussion, page 30, paragraph 4**  “Lastly, it appears that for the most part, the group with both depression and anxiety was more severely impacted both in terms of the number of outcomes and the strength of association. This finding is in keeping with prior studies which have found that those with both depression and anxiety experience worse outcomes (e.g., greater symptom severity, more disability, poorer work performance, etc.) compared to those with either depression or anxiety only or neither [53–55]. Perhaps these participants were experiencing a greater number of symptoms due to an additive effect from depression and anxiety.” |
| 3. Regarding the statistical analysis, the observation was made that there are differentiated effects by age groups, but in the regression analysis the authors only included the continuous variable, and it is not mentioned that non-linearities were evaluated. And the authors only responded that there were already many categorical variables.  Due to the above, I think that the article only provides information on prevalence, since the association analysis is still very incipient. | We have changed our logistic regression to a multinomial logistic regression that uses age groups (please see Table 3). |

| Reviewer Comment | Author Responses |
| --- | --- |
| **Reviewer 2** | We thank Reviewer 2 for their helpful and insightful comments on our manuscript. We have revised accordingly for each item. Please see details below. |
| 12. The new version included many recommendations from reviewers and is better than the previous one. However, it has an inconsistency between the introduction and analysis of the data. In the introduction it is indicated that the context can be a determinant of outcomes, but it is not explored. Only individual data is analyzed. The authors' explanation for not doing multilevel analysis is insufficient. It is suggested that the authors point out as a limitation that only individual variables and not contextual variables were explored, which can lead to a psychological fallacy (see Diez-Roux AV. Bringing context back into epidemiology: variables and fallacies in multilevel analysis. Am J Public Health 1988;88(2):216-22. | We thank the reviewer for raising this point. A line has been added to the limitations to indicate that only individual and not contextual variables were explored.  **Discussion, page 31, paragraph 5**  “Additionally, only individual and not contextual variables were explored, which may lead to an individualistic fallacy whereby an assumption is made that individual-level outcomes can only be explained by individual-level variables (1).”   (1) Diez-Roux AV. Bringing context back into epidemiology: variables and fallacies in multilevel analysis. Am J Public Health. 1998 Feb;88(2):216-22. doi: 10.2105/ajph.88.2.216. PMID: 9491010; PMCID: PMC1508189. |

We wanted to thank the editor and all of the reviewers again for their thoughtful review of our manuscript and for helping to strengthen it further.
